# Preparation, Characterization and Application of Multi-Mode Imaging Functional Graphene Au-Fe_3_O_4_ Magnetic Nanocomposites

**DOI:** 10.3390/ma12121978

**Published:** 2019-06-19

**Authors:** Wei Sun, Shaowen Huang, Siyu Zhang, Qi Luo

**Affiliations:** 1 School of Materials Science and Engineering, Nanchang University, Nanchang 330031, China; 5710116047@email.ncu.edu.cn (W.S.); siyuzh@163.com (S.Z.); 2 School of Civil Engineering, Guangdong Province Key Laboratory of Durability for Marine Civil Engineering, Shenzhen University, Shenzhen 518061, China

**Keywords:** graphene oxide, Au-Fe_3_O_4_ nanoparticles, targeting cancer cells, drug delivery

## Abstract

Nanomaterials extensively studied by nanotechnology scientists have been extensively applied in biomedicine, chemistry, physics and other fields nowadays. Magnetic nanoparticles, surpassing nano applications, are found to possess many advantages over nonmagnetic nanomaterials. Graphene oxide (GO), in particular, draws growing scholarly attention due to its large surface area, good water solubility and biocompatibility, rich surface functional group and easy-to-modify property. In this paper, we modify the Polyethylene mide (PEI) molecule on the surface of GO to increase its biocompatibility. The Au-Fe_3_O_4_ nanoparticles and folic acid molecules on the ligand make the resulting composite applicable both in magnetic resonance imaging and in cancer cell targeting. In addition, the π-π accumulation of doxorubicin used to load the anticancer drug can release the drug under the acid condition of the cancer cells, detect the cancer cells by fluorescence and realize the multi-mode detection of cancer cells.

## 1. Introduction

For recent decades, the number of cancer patients in the world has increased dramatically [1]. Cancer cell detection in the early stage of cancer and cancer cell targeting with sufficient accuracy to improve the selective toxicity are important topics in cancer treatment research [2]. Based on the idea that cancer cells and normal cells are in different environments, the technique of anticancer drug release to selectively kill cancer cells has drawn much attention from the academic and the public. Graphene oxide (GO) has attracted much attention from academia due to its excellent performance in absorbing and shielding electromagnetic wave [3,4]. Nandi et al. are the initiators on the surface of GO by ammonium persulfate as the initiator of free radical polymerization [5], and covalently combine with Poly(N-isopropylacrylamide) (PNIPAM). The prepared material is called GPNM. Compared with GO, this GPNM material improves its dispensability in the solution or cell medium, increases its biocompatibility and decreases its cytotoxicity. The oxygen containing functional groups on the surface of GO also makes GO have the characteristics of easy chemical modification, and the surface modifier can increase the biocompatibility of GO. Therefore, the graphene nano hybrid materials obtained by loading different types of metal nanoparticles on the surface of graphene can not only combine the inherent properties of GO and metal nanoparticles, but also give the new properties and functions of the composite, and expand the GO composite in physical [6], chemical [7], biological medicine [8] and other fields.

**Figure 1:**
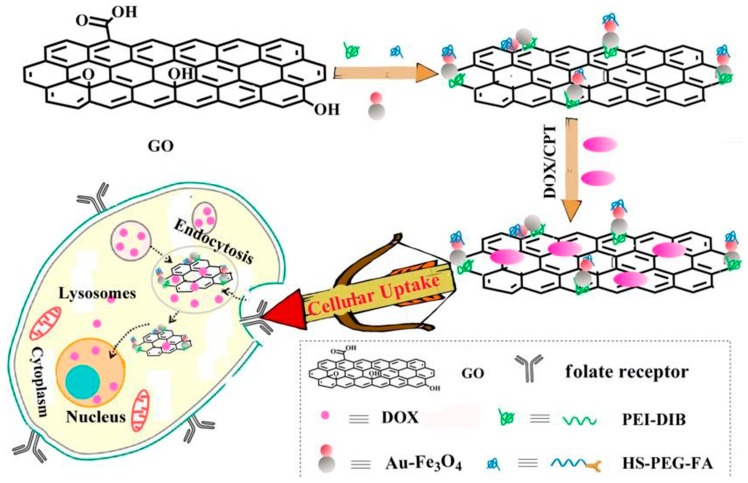
Preparation of graphene Au-Fe_3_O_4_ magnetic nanocomposite with multi-mode imaging function and its application in drug delivery.

Based on the above research background, we designed and synthesized a GO-based magnetic nanocomposite drug carrier, as shown in Figure 1. Firstly, amino groups on polyethylene mide (PEI) were covalently bound to the surface of GO by amide reaction. Then, 3,4-protocatechualdehyde containing phenol hydroxyl was covalently bound to the amino acid at the end of PEI. Since the oxygen on the phenol hydroxyl group is coordinated with the iron atoms of the iron oxide nano surface, and Fe_3_O_4_ has good electromagnetic characteristics [9] we used the Fe_3_O_4_ end of the Au-Fe_3_O_4_ nanoparticles to coordinate the phenolic hydroxyl groups. Based on the magnetic characteristics of Fe_3_O_4_, we can use this material in magnetic resonance imaging. Moreover, doxorubicin (DOX)itself has fluorescence emission at 595 nm. When DOX was adsorbed on the surface of GO, the fluorescence of DOX was quenched, and when the material was in the acid medium, DOX was separated from the GO surface and the fluorescence was restored to normal. Therefore, the material can also achieve multimode imaging in the detection of cancer cells, make up for the deficiency of single mode imaging, and make up for the two techniques of fluorescence imaging and magnetic resonance imaging to improve the location and diagnostic ability of small focus.

## 2. Experimental Reagents and Instruments

### 2.1. Experimental Reagents

The reagents used in the experiment are listed below in the Table 1. The reagents used later are shown in Table 1.

### 2.2. Experimental Instruments

The instruments used in the experiment are listed below in the Table 2. The instruments used later are shown in Table 2.

## 3. Experimentation

### 3.1. Preparation of Au-Fe_3_O_4_ Nanoparticles

The method of synthesizing Au-Fe_3_O_4_ nanoparticles is obtained by modifying according to the method described in the literature [10]. Firstly, 0.1 g HAuCl_4_.3H_2_O was added to the centrifugal tube and 10 mL oleamine and 10 mL naphthalene tetrahydride were added to dissolve it. After dissolving, the yellow solution was obtained. The 0.087 g Tetrabutylammonium bromide (TBAB) (1 mmol) was dissolved in a mixed solution of 1 mL oleamine and 1 mL four naphthalene. After the solid solution was completely dissolved, HAuCl_4_ solution was added quickly to obtain a purple red solution. The purple-red solution was stirred at room temperature for 1 h, and then 40 mL ethanol was added, centrifuged and dried. Finally, 5 nm Au nano-solid was collected.

The dry solid Au nanoparticles 20 mg were dissolved in 1 mL mixture of oleic acid, 2 mL oleamine and 20 mL 1-eighteen olefins. When the solid Au nanoparticles were completely dissolved, nitrogen was injected while stirring. After the air was basically exhausted, the temperature rose slowly to 120 °C. In order to ensure that the low boiling point solvent can be completely eliminated, we maintained 1 h at 120 °C in nitrogen atmosphere. Then the outlet valve was closed and 0.10 mL Fe(CO)_5_ was rapidly injected under the nitrogen cover. At the same time, the temperature rapidly raised to 300 °C and the timing started. The reaction was maintained for 25 min. After the reaction stopped, the heating was stopped, cooled under nitrogen protection to about 40 °C, then the lid was oxidized and isopropanol was added. After centrifuging the liquid, the collected solids were cleaned three times with a mixture of n-hexane and ethanol, and the product was eventually dispersed into n-hexane for preservation.

### 3.2. Preparation of 1a

GO solid 50 mg was dissolved in 30 mL dry Dimethyl sulfoxide (DMSO) solution and ultrasonic 2 h. Then dimethylaminopyridine (DMAP) (0.022 g), Hydroxybenzotriazole (HOBT) (0.052 g), ethylcarbodiimide hydrochloride (EDC·HCl) (0.072 g) were added to activate the carboxyl group on the surface of GO. In addition, 20 mg PEI was dissolved in 20 mL dried DMSO solution, and then added PEI solution to the activated GO solution, while ultrasonic dropping [11]. After the end of the drop, the light response at room temperature was 24 h. After the reaction was finished, ethanol was added to the above solution for washing centrifugation. Finally, the mixture of DMSO and ethanol was cleaned three times.

### 3.3. Preparation of 1b

1a (50 mg) prepared above, was dispersed in ethanol solution by ultrasonic dispersion of 1 h, and then 4 mg Diisobutylene (DIB)solid powder was taken and dissolved in 8 mL ethanol solution and added to in the ethanol solution of 1a [12]. At room temperature, the reaction was 24 h. After the reaction, the solution was centrifuged with petroleum ether, and finally washed with ethanol three times. The final product, 1b continued to disperse in the DMF solution.

### 3.4. Preparation of 1c

Twenty mg Au-Fe_3_O_4_ nanoparticles prepared above were dissolved in the 20 mL trichloromethane solution, and 8 mg HS-polyethylene glycol-NH_2_ (HS-PEG-NH_2_) and 2 mg HS-polyethylene glycol-FA (HS-PEG-FA) were added to the solution, making the Au end of the Au-Fe_3_O_4_ nanoparticles modified on the upper HS-PEG-NH_2_ and a small amount, making the material highly targeted cancer cells. Then, 1b was dispersed in 10 mL Dimethylformamide (DMF)solution and slowly added to dichloromethane 30 mL under ultrasonic conditions. The modified Au-Fe_3_O_4_ nanoparticles were dispersed in a mixed solution of DMF and dichloromethane volume ratio of 1: 3, 24 h was stirred at room temperature after 30 min. After the reaction ended with petroleum ether centrifugation, the final collected solid continued to be washed with the mixed solution of DMF and ethanol. The black solid were 1c, and the 1c was frozen and dried.

### 3.5. Preparation of 1d

Six mg 1c was dispersed in 18 mL DOX or 7-Ethyl-10-Hydroxy-Camptothecin (SN38) solution with different concentrations. After 30 min of ultrasound, the mixture was stirred overnight under shading at room temperature. The product was centrifuged at 13500 rpm for 15 min, diluted to 2 mL with different volumes of supernatant, and its ultraviolet absorption at 480 nm or 380 nm was measured [13,14]. The solids centrifuged are washed with DMSO for many times until the upper layer was colorless. The loading capacity of DOX loaded on nanocomposites was determined using Equation (1).

(1) $$\mathrm{Loading}\;\mathrm{capacity}=\frac{m_{\left(\mathrm{DOX}\right)}-m_{\left({\mathrm{DOX}}^{\prime }\right)}}{m_{\left(\mathrm{nanocomposites}\right)}}$$

where m_(DOX)_ is the initial amount of drug, m _(DOX^′^)_ is the amount of drug in the upper layer, and m_(nanocomposites)_ is the amount of nanocomposites added.

### 3.6. Drug Release Test

We prepared three types of solutions that are of pH 4.5, 7 and 8.6, respectively, with 1/15 mol/L Na_2_HPO_4_ and NaH_2_PO_4_ buffer, and 3 mL of the above two buffer solutions were taken respectively, and three portions of equal mass of the above 1d were dispersed in the phosphate buffer saline (PBS)buffer solution of the above three different pH respectively, and packed in the dialysis bag and soaked in the 7 mL corresponding to the dialysis bag. The PBS buffer solution of pH was kept in the shaking table at 37 °C. Then at different time points, the PBS buffer solution of the outer layer was taken 2 mL to measure the intensity of the UV absorption peak at the corresponding position. The release of the drug was calculated by the UV absorption value, and the solution after the test was then reversed to [15].

## 4. Results and Discussion

### 4.1. Morphological Changes of Au-Fe_3_O_4_ Nanoparticles on GO Surface before and after Modification

**Figure 2:**
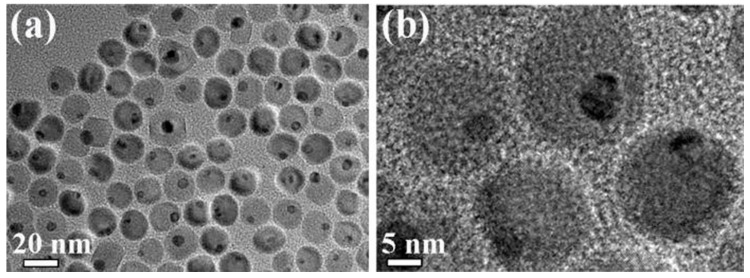
TEM diagram of Au-Fe_3_O_4_ nanoparticles: (**a**) High resolution map uniformly dispersed in n-hexane solution (**b**) High resolution graph.

Figure 2 shows that the Au-Fe_3_O_4_ nanoparticles are uniformly dispersed in the Transmission electron microscope (TEM) diagram of n-hexane solution. In particular, Figure 2a shows that the Au-Fe_3_O_4_ nanoparticles prepared by this high-temperature thermal decomposition method are homogeneous, and the edges of the Au nanoscale are in contact with the edges of Fe_3_O_4_ nanoparticles. The high-resolution map shows that the diameter of Au nanoparticles is about 5 nm, Fe_3_O_4_ nanoparticles is about 16 nm.

**Figure 3:**
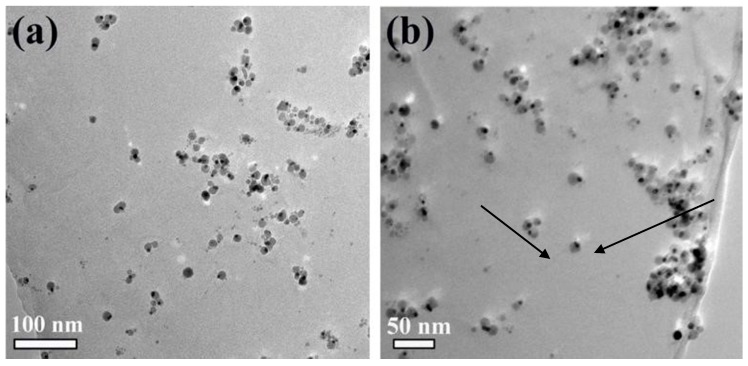
TEM diagrams of the Au-Fe_3_O_4_ nanoparticles modified on the surface of graphene oxide (GO) (**a**) scales are 100 nm; (**b**) scales are 50 nm.

After covalently combining with PEI on the GO surface, the 3,4-protocatechualdehyde is then connected with the Schiff base reaction, and the Au-Fe_3_O_4_ nanoparticles are covalently combined on the GO surface through the coordination of the phenolic hydroxyl group with the Fe atom. As shown in Figure 3, modifying Au-Fe_3_O_4_ nanoparticles with GO did not affect their morphology and size. The Au-Fe_3_O_4_ nanoparticles are scattered on the surface of GO.

**Figure 4:**
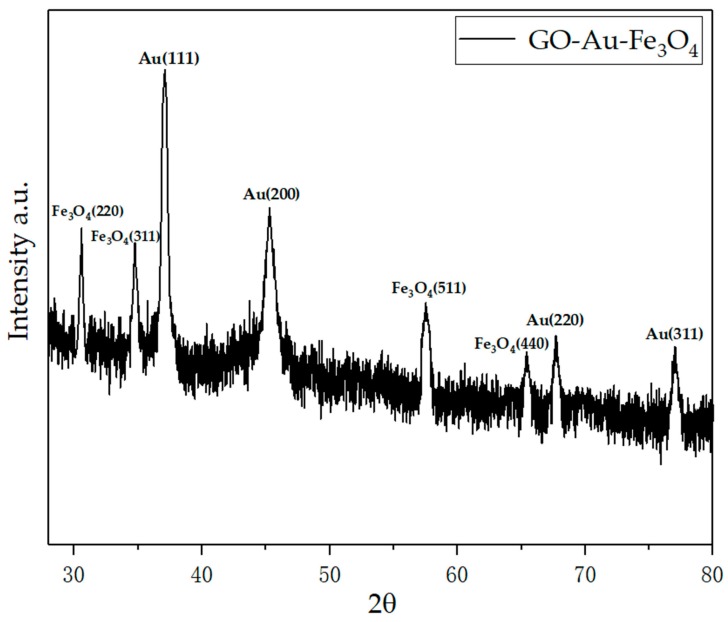
XRD pattern for GO-Au-Fe_3_O_4._

Figure 4 shows the X-ray diffraction (XRD) pattern of GO-Au-Fe_3_O_4_ nanocomposites, and it shows the presence of Fe_3_O_4_ and Au phases. 

### 4.2. Thermo Gravimetric Analysis of GO and GO-PEI

**Figure 5:**
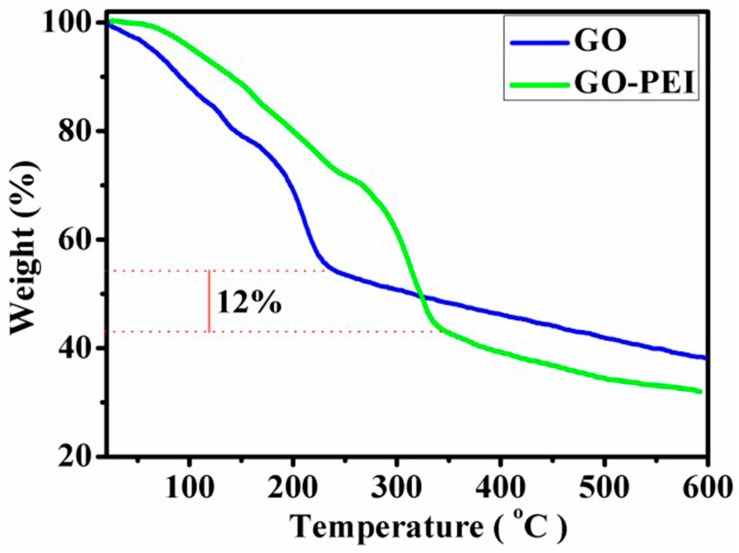
TGA diagrams of GO and graphene oxide- polyethylene mide (GO-PEI) in nitrogen atmosphere.

In order to study the thermal stability of GO and GO-PEI, two kinds of materials were analyzed by Thermal Gravimetric Analyzer (TGA), and Figure 5 shows the change of the weight loss curve of the sample in a nitrogen atmosphere with increasing temperature. GO showed thermal instability, and the weight of GO samples near the temperature of 230 °C lost nearly 45%. The loss of the weight was attributed to the decomposition of the unstable oxygen functional groups and the presence of a large number of oxygen functional groups on the GO surface. Compared with GO, the weight loss of GO-PEI is relatively small near 230 °C, which may be due to the covalent binding between the PEI and GO surface functional groups. The weight loss of GO-PEI in the interval of 245–600 °C is serious, mainly due to the decomposition of PEI and the decomposition of some oxygen - containing functional groups on the surface of the GO lamellar surface. If the thermal decomposition of 15% oxygen function groups in 245–600 °C is subtracted during the thermal decomposition of GO-PEI, it suggests that the content of PEI on GO-PEI is about 12%.

### 4.3. Infrared Analysis of Graphene-Based Au-Fe_3_O_4_ Magnetic Nanocomposites

**Figure 6:**
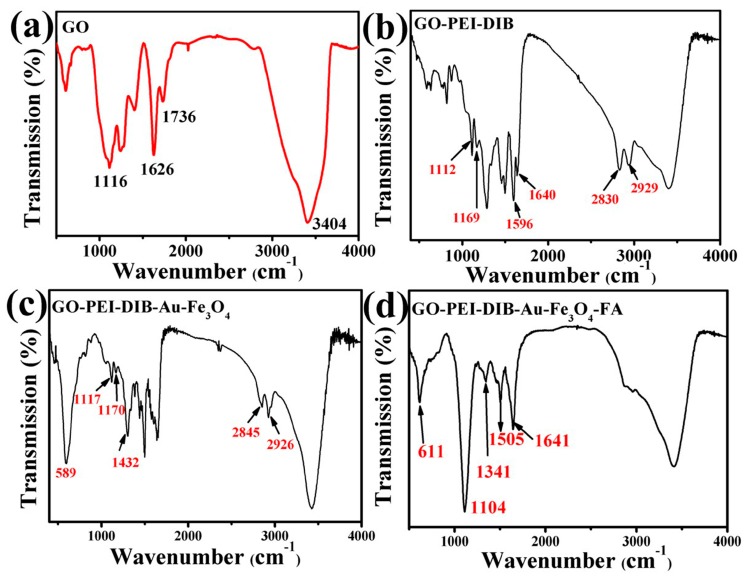
IR spectrum of (**a**) GO (**b**) 1B (**c**) 1C (**d**) 1D.

From the IR spectrum of GO, as shown in Figure 6a, the visible C=O peak of -COOH appears at 1736 cm^-1^, and a new peak appears at 1640 cm^−1^ in the diagram of 6b, because the -COOH on GO has an amide reaction to the amino group on the PEI, and the peak of the 1640 peak is the peak of the amide bond. At the same time, two distinct new peaks, 2845 and 2926 cm^−1^, belong to the symmetric and asymmetric stretching vibration peaks of CH2 in the PEI chain. In addition, the 1456 cm^−1^ is the C-N stretching vibration peak and 1596 cm^−1^ is the bending vibration peak of N-H. The emergence of these new peaks shows that PEI is successfully covalent on the surface of GO. The small peak at 1169 cm^−1^ is the phenolic hydroxyl peak on DIB, which also supports the successful connection of DIB. Figure 6c is shown as the infrared spectrum of 1C. Compared with Figure 6b, it is obvious that a distinct Fe-O vibration absorption peak appears at 589 cm^−1^, and the vibration absorption peak of C-O-Fe appears at 1117 cm^−1^ and the phenol hydroxyl peak at 1170 cm^−1^ is obviously weakened. The above evidence strongly proves the successful covalent binding of Au-Fe_3_O_4_. Figure 6d shows that FA belongs to the characteristic absorption peak of the benzene ring structure of the FA molecule at 1505 cm^−1^.

### 4.4. Magnetic Change of Au-Fe_3_O_4_ before and after Modification

**Figure 7:**
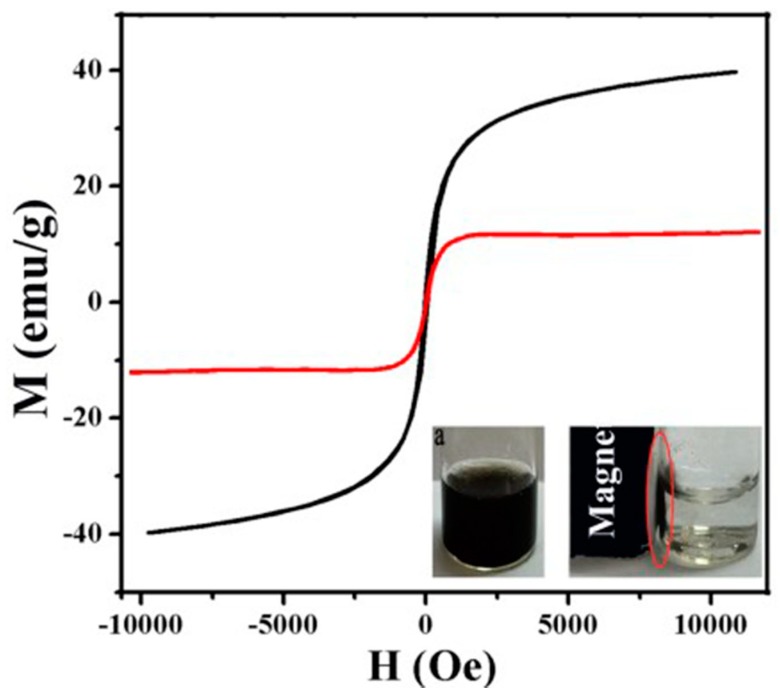
Hysteresis loops of graphene Au-Fe_3_O_4_ magnetic nanocomposites (red). Au-Fe_3_O_4_ hysteresis loop (black).

The magnetic saturation strength of unmodified Au-Fe_3_O_4_ nanoparticles is high, and its value can reach 40 emu/g (the black curve). The magnetic saturation strength (the red curve) of Au-Fe_3_O_4_ magnetic nanocomposites on the surface of graphene oxide by chemical modification is significantly reduced compared with that of the unmodified Au-Fe_3_O_4_ nanoparticles, and the magnetic saturation strength is 12 emu/g shown in Figure 6. Due to the magnetic properties of large molecular weight polymer modified graphene magnetic nanocomposites, the magnetic saturation strength of graphene Au-Fe_3_O_4_ magnetic nanocomposites is much lower than that of unmodified Au-Fe_3_O_4_ nanoparticles. As shown in Figure 7, compared with Au-Fe_3_O_4_ magnetic nanocomposites, the hysteresis of graphene Au-Fe_3_O_4_ magnetic nanocomposites was significantly reduced. Hysteresis loop had no hysteresis phenomenon and showed coercivity and residual magnetization close to 0 when there is no external magnetic field (H = 0). Therefore, graphene Au-Fe_3_O_4_ magnetic nanocomposites are superparamagnetic, as shown in Figure 7. When the external magnetic field is applied to graphene magnetic nanocomposites dispersed in aqueous solution, the composites can be rapidly separated from aqueous solution under the action of the applied magnetic field.

### 4.5. Graphene-Based Au-Fe_3_O_4_ Magnetic Nanocomposites and UV Analysis during Drug Loading

**Figure 8:**
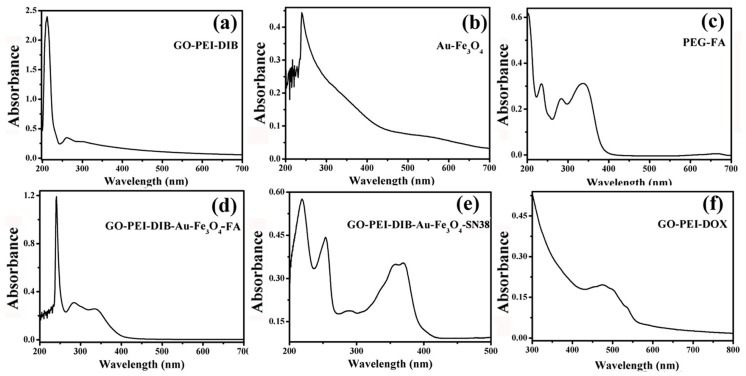
UV-Vis diagram of (**a**) Graphene oxide-Polyethylene mide-Diisobutylene (GO-PEI-DIB) (**b**) Au-Fe_3_O_4_ (**c**) polyethylene glycol-Folic acid (PEG-FA) (**d**) Graphene oxide-Polyethylene mide-Diisobutylene-Au-Fe_3_O_4_-Folic acid (GO-PEI-DIB-Au-Fe_3_O_4_-FA) (**e**) Graphene oxide-Polyethylene mide-Diisobutylene-Au-Fe_3_O_4_-7-Ethyl-10-Hydroxy-Camptothecin (GO-PEI-DIB-Au-Fe_3_O_4_-SN38) (**f**) Graphene oxide-Polyethylene mide-doxorubicin (GO-PEI-DOX).

Figure 8a, the UV curve of 1b, shows that there is a strong absorption peak around 230 nm. Usually, the structure of the large conjugated system will appear in the absorption peak at this place. For GO, the peak is generally regarded as a characteristic absorption peak of GO, which can prove the existence of GO. However, Figure 8b is the absorption peak of unmodified Au-Fe_3_O_4_ nanoparticles. Usually most of the nanoparticles have strong ultraviolet absorption peak at 280 nm. A strong UV absorption peak appears at 350 nm [16], while the FA absorption peak at 350 nm in the UV absorption diagram of Figure 8c PEG-FA is obvious. Characteristic peaks of Au-Fe_3_O_4_, GO and FA can be seen in the ultraviolet image of 8dGO-PEI-DIB-Au-Fe_3_O_4_-FA, indicating that Au-Fe_3_O_4_ and FA have been successfully attached to the surface of GO. In the UV absorption curve of GO-PEI-DIB-Au-Fe_3_O_4_/SN38 in Figure 8e, the two characteristic absorption peaks of SN38 at 367 nm and 383 nm are clearly visible, which indirectly indicates that SN38 is adsorbed on the GO surface. The characteristic absorption peaks of 4-8f GO-PEI/DOX UV-Vis spectra near 480 nm are also clearly visible, which indirectly proves that DOX is adsorbed on the GO surface.

### 4.6. Loading Capacity of Graphene-Based Au-Fe_3_O_4_ Magnetic Nanocomposites for DOX

**Figure 9:**
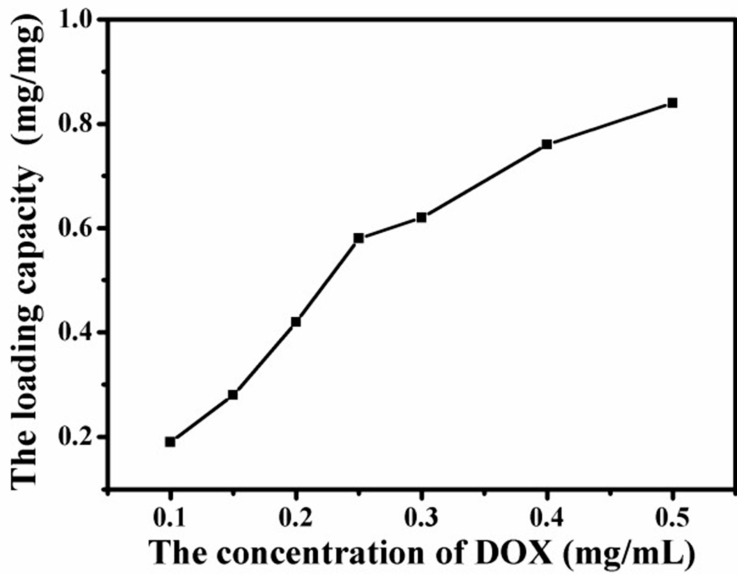
Change curves of drug loading of graphene Au-Fe_3_O_4_ magnetic nanocomposites with the increase of doxorubicin (DOX) concentration.

Due to the Amino and several hydroxyl groups on DOX, the –OH and –COOH groups on the graphene sheet can form a strong hydrogen-bonding interaction with –OH and –NH_2_ groups in DOX. Therefore, DOX was noncovalently loaded on graphene Au- Fe_3_O_4_ magnetic nanocomposites simply by mixing them in aqueous solution with the aid of slight sonication [17]. The loading capacity of DOX on GO was determined by the UV spectrum at 480 nm, which was calculated by the difference of DOX concentrations between the original DOX solution and the supernatant solution after loading [18]. The curves shown in Figure 9 describe the load of graphene Au-Fe_3_O_4_ magnetic nanocomposites with the change of DOX concentration. The diagram suggests that with the increase of DOX concentration, the load of graphene Au-Fe_3_O_4_ magnetic nanocomposites increases gradually. When the concentration of DOX reaches 0.5 mg/mL, the load value reaches 0.84 mg/mg load. From the curve trend, the load of DOX on graphene Au-Fe_3_O_4_ magnetic nanocomposite has not yet been saturated. Therefore, the saturated load of graphene Au-Fe_3_O_4_ magnetic nanocomposites loaded with DOX remains to be studied. Thus, as GO has a similar high specific surface area to graphene [19], and its large PI conjugate plane structure is special, the graphene oxide has a great potential application value in the nano drug carrier [17].

### 4.7. Release of DOX Loaded Graphene-Based Au-Fe_3_O_4_ Magnetic Nanocomposites under Different pH Conditions

**Figure 10:**
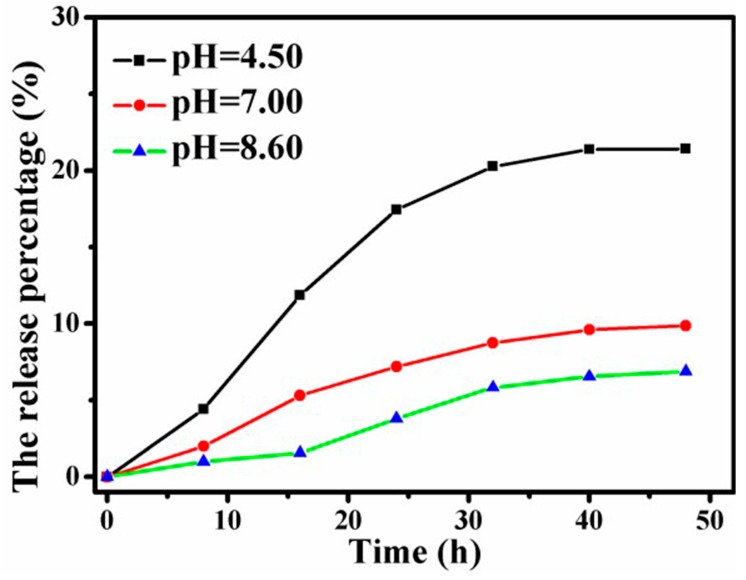
Relationship between DOX release components and time under different pH conditions.

In order to simulate the release state of anticancer drugs in the human body environment and cancer cells loaded with graphene Au-Fe_3_O_4_ magnetic nanocomplex load, the release of anticancer drugs is simulated in three PBS buffer solutions of pH = 4.5, 7 and 8.6, respectively. The change of the percentage of drug release with time is shown in Figure 10.

As can be seen from Figure 10, the release percentage of DOX can reach 21% after 48 h release in pH = 4.5 case. In the PBS buffer solution of pH = 7.0, the release percentage of the 48 h drug release is about 10%. The release of the drug is the lowest under the condition of pH = 8.6, and the release amount of the drug is less than 10%. The release curves also show that the 100 components released by the first 32 h drugs are gradually increasing with the duration of the release, until 32 h, the release of the drug basically reaches the platform and the rate of increase begins to decrease. In addition, the release of anticancer drugs under the acidic condition is about three times the amount of the drug release under the alkaline condition, suggesting that the acid condition is more beneficial to the release of the anticancer drug DOX. The reason is that DOX contains amino and hydroxyl groups, and the hydrophilicity and water solubility of DOX are significantly enhanced under acidic conditions. The release of pH - dependent drugs plays an important role in the clinic. The acidity of endosomes or lysosomes or the acid environment of the tumor tissue cells is beneficial to the release of drugs, which will have an important effect on anticancer and antitumor biological applications. However, considering the influence of temperature on nanocomposites, the nanocomposites released drug faster at 43 °C than at 25 °C in vitro release experiment [20]. For zeta potential, the zeta potential was in the range of 28.9 ± 3.5 and 50.8 ± 3.9 mV. These values suggest that the prepared nanoparticles systems are stable. This positive charge is alluring so as to avoid particles aggregation and to stimulate electrostatic interaction with the overall negative charge of the cell membrane. Therefore, positively charged nanoparticles are a perfect option for the preparation of drug delivery systems [21].

### 4.8. Loading Capacity of Graphene-Based Au-Fe_3_O_4_ Magnetic Nanocomposites for SN38

**Figure 11:**
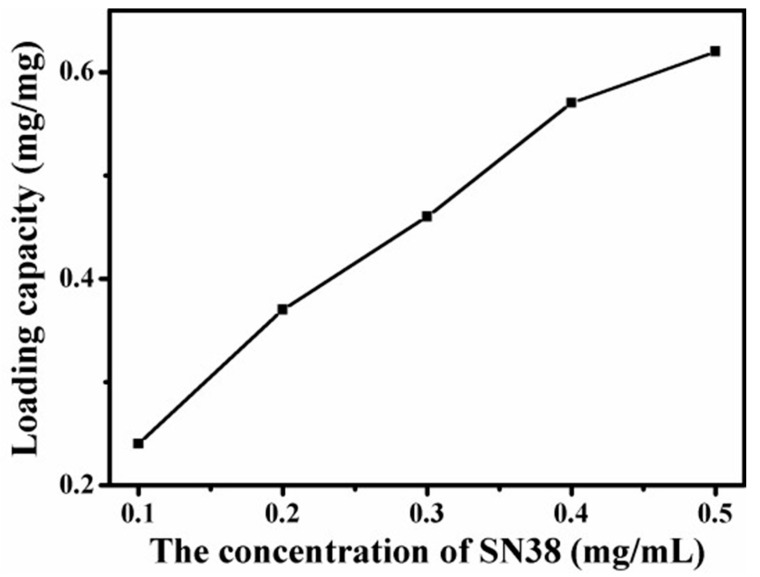
Curves of drug loading with graphene Au-Fe_3_O_4_ magnetic nanocomposites varying with 7-Ethyl-10-Hydroxy-Camptothecin (SN38) concentration.

Loading of SN38 on the surface of graphene Au-Fe_3_O_4_ magnetic nanocomposite is achieved by simple ultrasonic and mixed agitation. The curve shown in Figure 11 depicts the relationship between the loading of graphene Au-Fe_3_O_4_ magnetic nanocomposites and the concentration of SN38. The diagram shows that with the increase of the SN38 concentration, the load of graphene Au-Fe_3_O_4_ magnetic nanocomposites increases gradually. When the concentration of SN38 reaches 0.5 mg/mL, the load value reaches 0.6 mg/mg load. The curve shows that the load of SN38 on graphene Au-Fe_3_O_4_ magnetic nanocomposites is not yet saturated. Therefore, the saturated load of graphene Au-Fe_3_O_4_ magnetic nanocomposites loaded with SN38 remains to be studied. Compared with the adsorption of DOX, the adsorption amount of SN38 is less than that of DOX. The reason may be because the molecular structure of the two is different. DOX is hydrophilic because of its hydroxyl and amino groups on its surface, and SN38 is a hydrophobic drug with few oxygen functional groups on the surface, generating water and others often. The solubility in organic solvents is also very low. Therefore, when two anticancer drugs are mixed with graphene Au-Fe_3_O_4_ magnetic nanocomposites through π-π stacking and hydrophobic interaction, the adsorption capacity is different.

### 4.9. Release of SN38 Loaded Graphene-Based Au-Fe_3_O_4_ Magnetic Nanocomposites under Different pH Conditions

**Figure 12:**
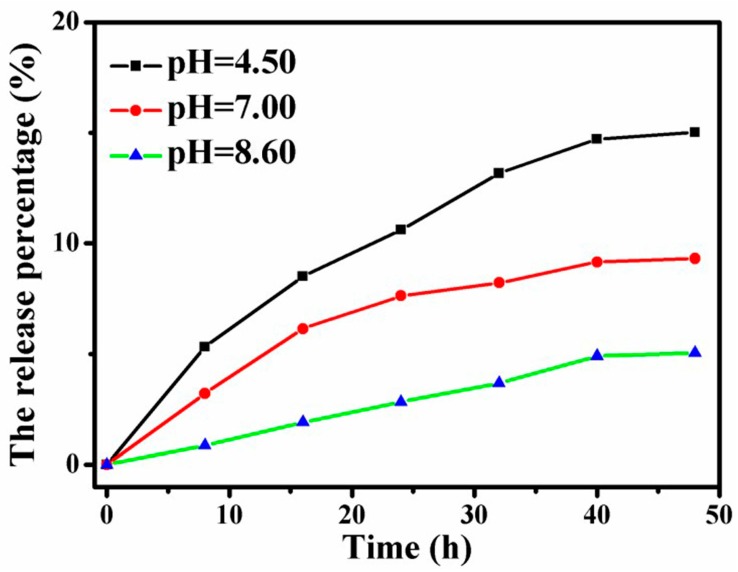
Relationship between the release of SN38 and the time dependence of pH under different conditions.

In order to simulate the release state of anticancer drugs in the body environment and cancer cells of the Au-Fe_3_O_4_ magnetic nanocomplex loaded with graphene, as shown in Figure 12, the release of anticancer drugs is simulated in three PBS buffer solutions of pH = 4.5, 7 and 8.6, respectively. The diagram shows that the release percentage of SN38 can reach about 15% after 48 h releases in pH = 4.5. The release percentage of the 48 h drug released in the PBS buffer solution of pH = 7.0 is about 9%, and the release of the drug is the lowest under the condition of pH = 8.6. Under the condition of pH = 8.6, the release of drug is the lowest. The release curve shows that the 100 components released by the first 32 h drugs gradually increase with time [22,23,24]. After 32 h, the release amount of the drug reaches the platform with a slower increase rate. Compared values of drug release percentages under acid and alkaline conditions, it is obvious that acidic conditions are more favorable for the release of SN38. Under the influence of hydrophobic structure, the drug release effect of SN38 is worse than that of DOX. It is noteworthy, however, that the drug release from this drug-loaded material is pH-responsive and sustained, and ultimately achieves controlled and sustained release.

## 5. Conclusions

Polyethylene mide (PEI) is covalently modified to GO surface, and 3, 4- protocatechualdehyde is attached to -NH_2_ by Schiff base reaction. By the coordination of its phenol hydroxyl oxygen with the Fe atom of Fe_3_O_4_ nanoparticles, the Au-Fe_3_O_4_ nanoparticles are covalently bonded to the GO surface through the coordination of Fe_3_O_4_ and phenol hydroxyl oxygen. At the same time, Au nanoparticles can bind to HS-PEG-FA by the properties of SH ligand, so that the GO based Au-Fe_3_O_4_ magnetic nanocomposites can bind to the cancer cells highly expressed by FR. In addition, using the π-π accumulation of GO and anticancer drugs on the anticancer drug greatly improves the concentration of anticancer drugs in cancer cells to achieve better therapeutic effect. In the detection of cancer cells, because of the superparamagnetic properties of Fe_3_O_4_ nanoparticles, magnetic resonance imaging can be performed under the external magnetic field, and the anticancer drugs loaded on the GO surface such as DOX or SN38 release drugs in the acid environment of the cancer cells. The fluorescence of these anticancer agents itself will also be quenched by the original. The state is restored, so fluorescence imaging can be performed simultaneously. Combining the advantages of magnetic resonance imaging and fluorescence imaging, the composite material is expected to greatly improve the sensitivity and accuracy of cancer cell detection.

## Figures and Tables

**Table 1:** Experimental reagents.

Reagents	Specifications	Manufacturer
Ferric acetylacetonate	A.R.	Shanghai Aladdin Biochemical Technology Co., Ltd, Shanghai, China
N-(4-Pyridyl)dimethylamine	A.R.	Shanghai Aladdin Biochemical Technology Co., Ltd, Shanghai, China
3,4- protocatechualdehyde oleamine	A.R.	Sigma–Aldrich, St. Louis, MO, USA
NHydroxybenzotrizole	A.R.	Sigma–Aldrich, St. Louis, MO, USA
Dopamine	A.R.	Sigma–Aldrich, St. Louis, MO, USA
Thioglycolic acid; mercaptoacetic acid	A.R.	Sigma–Aldrich, St. Louis, MO, USA
Anthranilic acid	A.R.	Sigma–Aldrich, St. Louis, MO, USA
Folic acid	A.R.	Sigma–Aldrich, St. Louis, MO, USA
Isatoic anhydride	A.R.	Sigma–Aldrich, St. Louis, MO, USA

**Table 2:** Experimental instrument.

Instrument	Model	Manufacturer
Fluorescence spectrophotometer	F4500	Hitachi, Ltd, Tokyo, Japan
Transmission electron microscope	HT7800	Hitachi, Ltd, Tokyo, Japan
X-ray Photoelectron Spectrometer	ESCALAB 250 XI	Thermo Fisher Scientific, Hillsboro, OR, USA
Fourier transformation infrared spectrometer	IR-960	Tianjin Ruian Technology Co., Ltd, Tianjin, China
Ultraviolet visible spectrophotometer	UV 1750	Shimazduo, Kyoto, Japan
X-ray diffractometer	D8 Advance	BRUKER AXS GMBH, Karlsruhe, Germany
